# Weak anti-SARS-CoV-2 antibody response is associated with mortality in a Swedish cohort of COVID-19 patients in critical care

**DOI:** 10.1186/s13054-020-03362-y

**Published:** 2020-11-06

**Authors:** Sana Asif, Robert Frithiof, Miklos Lipcsey, Bjarne Kristensen, Kjell Alving, Michael Hultström

**Affiliations:** 1 Anesthesia and Intensive Care Medicine, Department of Surgical Sciences, Uppsala University Hospital, Uppsala University, Entrance 78, etg 1, 75185 Uppsala, Sweden; 2 Hedenstierna Laboratory, CIRRUS, Anesthesiology and Intensive Care, Department of Surgical Sciences, Uppsala University, Uppsala, Sweden; 3 Thermo Fisher Scientific, Alleröd, Denmark; 4 Department of Women’s and Children’s Health, Uppsala University, Uppsala, Sweden; 5 Integrative Physiology, Department of Medical Cell Biology, Uppsala University, Uppsala, Sweden

To the editor,

Profiling of the antibody responses to SARS-CoV-2 may be crucial to understand the immunological reaction and to design successful treatment strategies. Studies have confirmed the development of a typical antibody response to an acute viral infection in COVID-19 patients [1]. A robust generation and a dynamic pattern of IgA, IgM and IgG antibodies can be detected 2–3 weeks following the first symptoms of COVID-19 [2, 3]. An early robust antibody response in patients hospitalized with severe COVID-19 was reported in survivors, versus a weak antibody production in non-survivors [4, 5]. However, antibody responses to SARS-CoV-2 in critically ill patients is largely unknown.

We investigated the antibody response to SARS-Cov-2 Spike-1 protein in adult patients (*n* = 19) admitted to the intensive care unit (ICU) at a tertiary care hospital in Uppsala, Sweden. Plasma samples were collected at two different time points; (a) early, day 0–3 and (b) late, day 10–13, and concentrations of IgA, IgG and IgM antibodies were quantified by FluoroEnzymeImmunoassay (FEIA), Phadia AB, Uppsala, Sweden.

The median age of our cohort was 57 years, and 93% were males. The most common co-morbidities were obesity (93%), hypertension (42%) and diabetes mellitus type-2 (32%). The median COVID-19 day at ICU admission was 10 (6–14) days, the median length of ICU stay was 18 (11–38) days, and 30 days mortality was 21% (Table 1). In our cohort, a IgA, IgG and IgM antibody response could be detected as early as day 0–3 post-ICU admission, and this increase in antibodies was persistent up to day 10–13 (Fig. 1). A significant change in antibody concentrations over time was detected in patients who survived till day 30 in comparison with those who did not (Fig. 1). No associations were seen between antibody levels and patient age, or any other clinical or laboratory parameters. At both early and late timepoints, plasma concentrations of IgA, IgG and IgM antibodies tend to be higher in patients who survived compared to those who had died at 30 days (Fig. 1). This suggests that SARS-CoV-2 antibody response, similar to other virus illnesses, is important for virus protection and recovery. A limitation of the present dataset is the relatively low number of patients.

**Table 1:** Clinical characteristics of COVID-19 patients (*n* = 19) with at least 10 days length-of-stay in intensive care unit (ICU)

Variables	
Cohort characteristics	Median (range)
Age, years, median (range)	57 (26–76)
Male, *n* (%)	18 (93)
COVID-19 days, median (range)	10 (6–14)
Length-of-stay in ICU, median (range)	18 (11–38)
30-day mortality, *n* (%)	4 (21)
Comorbidities	*n* (%)
Obesity (BMI > 25)	18 (93)
Diabetes mellitus	6 (32)
Hypertension	8 (42)
Pulmonary disease	4 (21)
Cardiovascular disease	3 (16)
Clinical parameters	Median (range)
Fever (> 38 °C), *n* (%)	15 (79)
Mean arterial pressure, mmHg, median (range)	94 (68–137)
Heart rate, min^−1^, median (range)	92 (67–116)
Respiratory rate, min^−1^, median (range)	31 (15–50)
SAPS3 score, median (range)	49 (39–63)
Laboratory parameters	
SOFA score	7 (3–9)
CRP mg/L	241 (131–476)
Ferritin μg/L	676 (104–3960)
Lactate mmol/L	1.1 (0.8–1.7)

*SAPS3* simplified acute physiology score-3, *SOFA score* sequential organ failure assessment score, *CRP* C-reactive protein

**Fig. 1:**
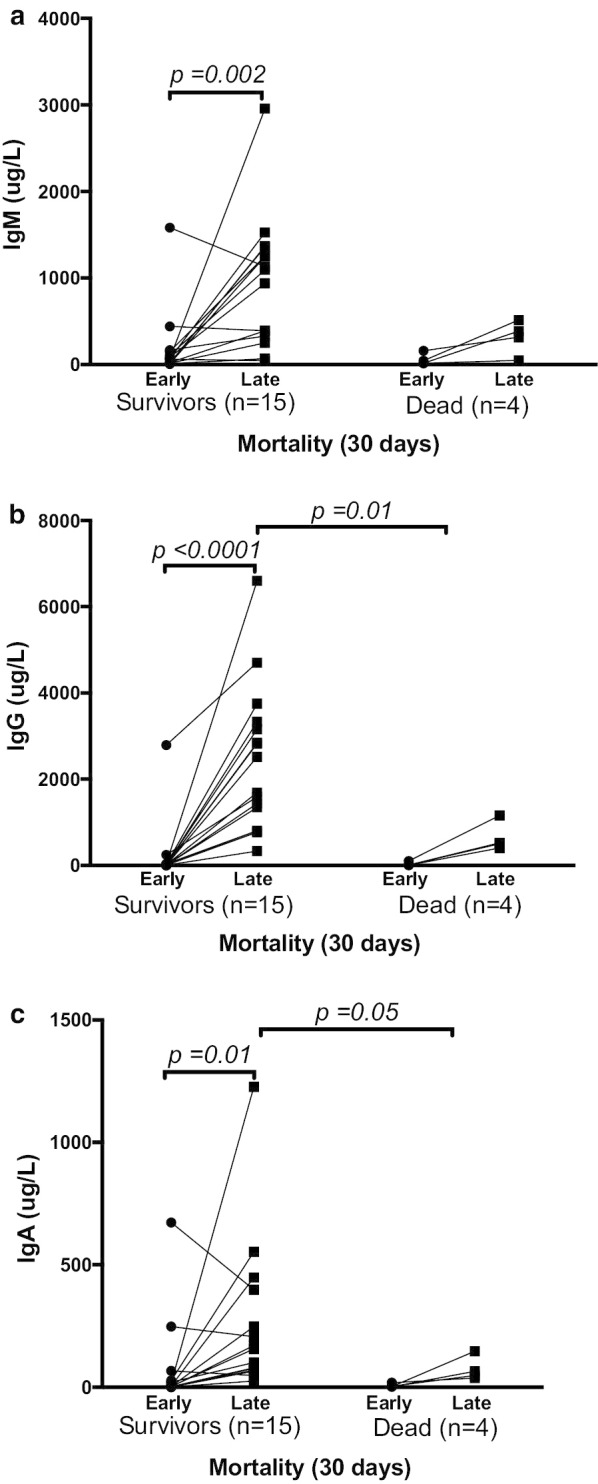
Plasma concentrations of IgM (**a**), IgG (**b**) and IgA (**c**) antibodies measured on ICU day 0–3 (early) and on ICU day 10–13 (late), in patients who survived (*n* = 15) of COVID-19 versus those who died (*n* = 4) within 30 days. Each data point on the graph represents individual values, differences were considered statistically significant when *P* < 0.05. Change over time within groups was determined by Wilcoxon signed rank test and between groups were determined by Mann–Whitney test. Plasma concentrations of all three antibody isotypes changed over time and were significantly higher on day 10–13 for IgG and IgA in patients who survived COVID-19 than in those who died

To our knowledge, this study provides the earliest evidence that an early and potent antibody response may contribute to infection clearance and improved prognosis in patients critically ill with COVID-19.

## Data Availability

Data in the current study are available from the corresponding author on reasonable request.
